# Delphi Consensus Statement on the Role of Probiotics in the Treatment of Atopic Dermatitis

**DOI:** 10.7759/cureus.64583

**Published:** 2024-07-15

**Authors:** Jayakar Thomas, Maleeka Sachdeva, Sandipan Dhar, Anil Ganjoo, Bela Shah, Deepika Pandhi, Koushik Lahiri, Rashmi Agarwal, Soumya Jagadeesan, Pradeep Mane, Rathish Nair, Krishnaprasad R Korukonda

**Affiliations:** 1 Dermatology, Jayakar Thomas Skin Care Centre, Chennai, IND; 2 Dermatology, Sachdev Clinics, Chandigarh, IND; 3 Dermatology, The Skin Clinic, Kolkata, IND; 4 Dermatology, Skinnovation Clinics, New Delhi, IND; 5 Dermatology, Byramjee Jeejeebhoy Medical College, Ahmedabad, Ahmedabad, IND; 6 Dermatology, University College of Medical Sciences and Guru Teg Bahadur Hospital, Delhi, IND; 7 Dermatology, Apollo Gleneagles Hospital, Kolkata, IND; 8 Dermatology, Meenakshi Cosmoderma Care, Bengaluru, IND; 9 Dermatology, Amrita Institute of Medical Sciences and Research Center, Ernakulam, IND; 10 Medical Strategic Affairs, Torrent Pharmaceuticals Ltd., Ahmedabad, IND

**Keywords:** immunomodulation, th2, th1, treg cells, lactobacillus rhamnosus gg, probiotic, atopic dermatitis

## Abstract

Background

Atopic dermatitis (AD) is a chronic inflammatory skin disease characterized by intense itching and recurrent eczematous lesions. Important factors in the etiopathogenesis of AD include genetic predisposition, epidermal barrier dysfunction, immune dysregulation, and gut and skin dysbiosis. Probiotics could be a potential preventive strategy for allergies including AD through immune system modulation as well as enhancement of the epithelial barrier integrity. To further understand the role of probiotics in the management of AD, a Knowledge, Attitude, and Practices (KAP) survey was conducted.

Materials and methods

A steering committee comprising nine experts formulated consensus recommendations on the role of probiotics in the management of AD and associated flare-ups through the use of the Knowledge, Attitude, and Practices questionnaire while analyzing literature reviews and responses from a national panel consisting of 175 members. The evidence strength and quality were evaluated based on the Agency for Healthcare Research and Quality (AHRQ) criteria. The acceptance of expert opinions as recommendations was considered upon receiving an endorsement from ≥70% of the panelists, as indicated by a Likert scale.

Results

The national panel emphasized that the improvement in nutritional status, immunomodulatory properties, and beneficial effects on the gastrointestinal (GI) tract and skin support the use of probiotics in AD. The panel agreed that probiotics should be a part of the complementary therapy in the management of AD and associated flare-ups. Mostly, a probiotics supplementation duration of eight to 12 weeks is preferred by dermatologists. Probiotics, when used as an adjuvant therapy, may serve as a strategy to reduce steroid usage or maintenance therapy in high-risk cases with flares.

Conclusion

A Delphi-mediated KAP response provides a real-life approach to the use of probiotics in the management of AD. It suggests that probiotics could be useful as an adjuvant therapy in the management of AD and associated flare-ups when used along with traditional treatment.

## Introduction

Atopic dermatitis (AD) is a chronic inflammatory skin disease characterized by intense pruritus and recurrent eczematous lesions [1]. Globally, it affects up to 20% of children and up to 10% of adults [2]. The pathogenesis of AD involves multiple factors like genetic predisposition, epidermal barrier dysfunction, systemic and local immune dysregulation, and gut and skin dysbiosis [3,4].

The AD management in childhood is challenging. Topical corticosteroid (TCS) administration might control the symptoms, especially in children with mild and moderate eczema. However, the occurrence of relapses is common. Moreover, prolonged and excessive use of TCSs implies a risk of systemic side effects and may cause skin atrophy, striae, rosacea, perioral dermatitis, acne, and purpura [5,6].

Evidence suggests that AD derives from a T-cell imbalance with the predominance of Th2 differentiation of naïve CD4+ T cells, which results in a greater production of IL-4, IL-5, and IL-13, which could be locally affected both by the activation of IgE and in eosinophils. Recently, it has been reported that probiotics could be a potential preventive strategy for allergies including AD through immune system modulation through the rebalancing of the Th1 and Th2 response as well as the enhancement of the epithelial barrier integrity [3,7].

The WHO has defined probiotics as live microorganisms, which when administered in adequate amounts, confer a health benefit on the host [8]. Lactobacillus and Bifidobacterium are the most commonly used probiotic strains.

Probiotics can be used as an adjuvant therapy in patients with moderate to severe AD [9]. Recently, a meta-analysis of 17 randomized, placebo-controlled trials that included AD patients under the age of 18 years showed the beneficial effect of probiotics on reducing the severity of AD [10].

To further assess the scope of, potential of, and contemporary practices for the management of AD with probiotics in real-world settings of India, this Knowledge, Attitude, and Practices (KAP) survey was conducted.

## Materials and methods

A KAP survey questionnaire was designed to explore the role of probiotics in treating AD within the Indian context. To ensure its validity, a panel of 10 experts, comprising practicing dermatologists experienced in developing consensus statements, undertook the validation process. The meeting for the same was held in August 2023. The KAP survey was distributed to 175 healthcare professionals (HCPs) across India specializing in AD management, and they were asked to provide their responses within 30 days.

In December 2023, a second round of meetings took place, centering on the examination of responses, literature review, and the development of consensus recommendations regarding the role of probiotics in AD management. The steering panel, comprising 10 experts, reviewed the literature and developed clinical recommendations. The establishment of the level of evidence (LoE) was achieved through an exhaustive review of publications accessible in indexed databases such as PubMed and Google Scholar using keywords such as Probiotics, Atopic Dermatitis, Atopic Flare-ups, Lactobacillus rhamnosus GG, Eczema, and Immune Dysregulation.

**Figure 1:**
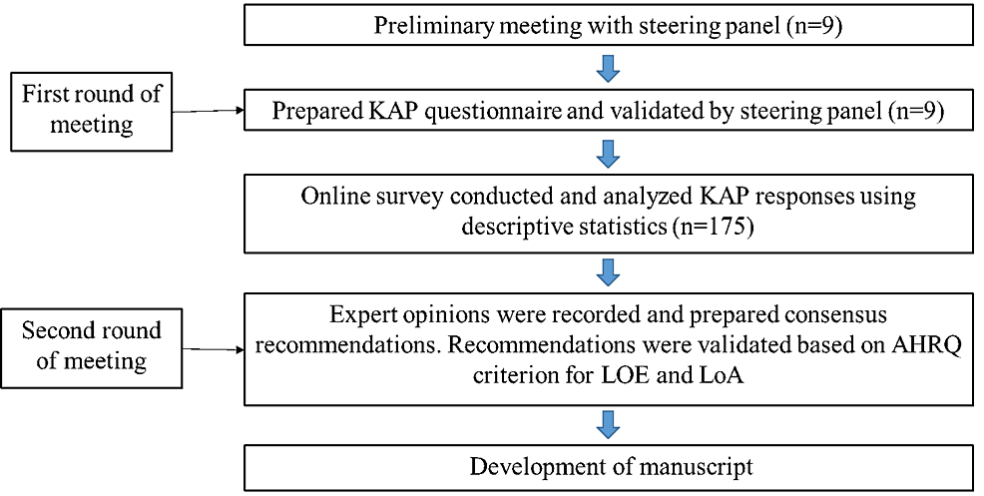
Study procedure flow chart KAP: Knowledge, Attitude, and Practices; AHRQ: Agency for Healthcare Research and Quality; LOE: level of evidence; LoA: level of agreement

The resulting consensus recommendations, derived from a problem-based clinical assessment approach, were used to consider the strength and quality of the evidence in accordance with the criteria set by the Agency for Healthcare Research and Quality (AHRQ), coupled with the general expert opinion shared by the panel. LoEs were assigned to various study types, categorizing meta-analyses, randomized, case-cohort longitudinal, cross-sectional case-control, and case reports as Level I, II, III, IV, and V, respectively. The acceptance of expert opinions as recommendations were considered upon receiving an endorsement from ≥70% of the panelists, as indicated by a Likert-type scale score (1: Strongly Disagree, 2: Disagree, 3: Neutral, 4: Agree, and 5: Strongly Agree) during the meeting. Descriptive statistical analyses, encompassing mean, median, and proportion assessments, were conducted for each response by using the Microsoft Excel 2016 version (Microsoft Corporation, Redmond, Washington, United States). A detailed study procedure flow chart is depicted in Figure 1.

## Results

The study aimed to assess the current usage of oral probiotics in the management of AD by gathering insights from 175 practicing dermatologists. To achieve this, a KAP-based questionnaire was employed. This survey tool was designed to capture detailed information on the perceptions, experiences, and clinical practices of these healthcare professionals regarding the use of probiotics, particularly Lactobacillus rhamnosus GG (LGG), in treating AD. The participating dermatologists were located in various regions across India, which ensured a diverse and representative sample. The collected responses were meticulously compiled and subjected to a comprehensive analysis to identify trends, common practices, and potential areas for improvement in the application of probiotics in dermatology. These questions assessed various aspects of the clinical use of probiotics in managing AD. They explored the perceived role and timing of probiotics, the effectiveness of specific strains like LGG, and the recommended duration of treatment. The questions also evaluated beliefs about the broader benefits of probiotics, such as improving nutritional status, immune response, and overall gastrointestinal and skin health. Additionally, they investigate the acceptance of probiotics as a complementary therapy and their potential to reduce the use of topical immunosuppressants in AD treatment. Table 1 shows the structured questionnaire.

**Table 1:** KAP questionnaire with responses on the clinical role and positioning of probiotics in AD AD: atopic dermatitis; KAP: Knowledge, Attitude, and Practices

No.	Structured questionnaire	Response	N, %
1	According to your clinical practice, where would you like to place probiotics in the management of AD?	Prevention of AD	107, 61%
		Treatment of AD	68, 39%
2	Probiotics in the right concentration are effective in the management of flare-ups in AD.	Agree	152, 87%
		Neutral	23, 13%
3	Lactobacillus rhamnosus GG strain is more effective for the prevention of AD than other commercially available probiotics.	Agree	145, 83%
		Neutral	30, 17%
4	According to your clinical practice, how long will you prescribe probiotics in AD?	two weeks	5, 3%
		two to four weeks	14, 8%
		four to six weeks	32, 18%
		eight to 12 weeks	73, 42%
		>12 weeks	51, 29%
5	The improvements in nutritional status, nutrient digestion, and specific and non-specific immune response, and the beneficial effects on the gastrointestinal tract and skin, support the use of probiotics in patients with AD.	Agree	149, 85%
		Neutral	26, 15%
6	Probiotics should be a part of the complementary therapy for the management of AD and associated flare-ups.	Agree	133, 76%
		Neutral	37, 21%
		Disagree	5, 3%
7	Probiotics reduce the usage of topical immunosuppressants (topical corticosteroids, TCSs) in AD.	Agree	136, 78%
		Neutral	32, 18%
		Disagree	7, 4%

## Discussion

**Figure 2:**
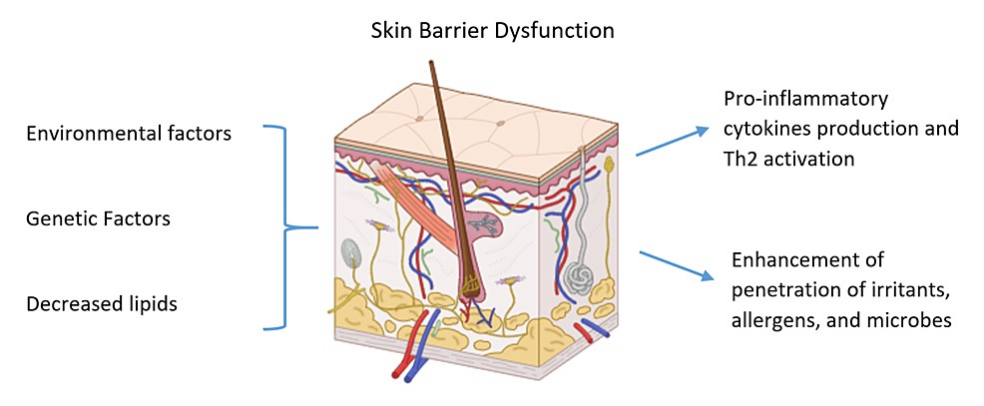
Pathophysiology of atopic dermatitis Adapted from Tomáš Kebert & umimeto.org, 2020 [13]; CC BY-SA 4.0

Skin barrier abnormalities are considered as the initial step in the pathogenesis of AD. Barrier defects may be linked to lack of filaggrin, altered skin microbiome, altered composition of lipids in stratum corneum, and deficiency of antimicrobial peptides (AMPs). A Th2 predominance characterizes the immune response in AD, leading to chronic inflammation. This Th2-skewed response, independent of skin barrier abnormalities, perpetuates the disease. Chronic inflammation results in epidermal hyperplasia and immune cell infiltration, worsening the condition. While skin barrier abnormalities initiate AD, Th2 predominance sustains it. Thus, effective management of AD requires addressing both skin barrier defects and immune system responses. Skin barrier dysfunction leads to chronic inflammation with epidermal hyperplasia and cellular infiltrates [11,12]. Figure 2 describes the pathophysiology of AD.

**Figure 3:**
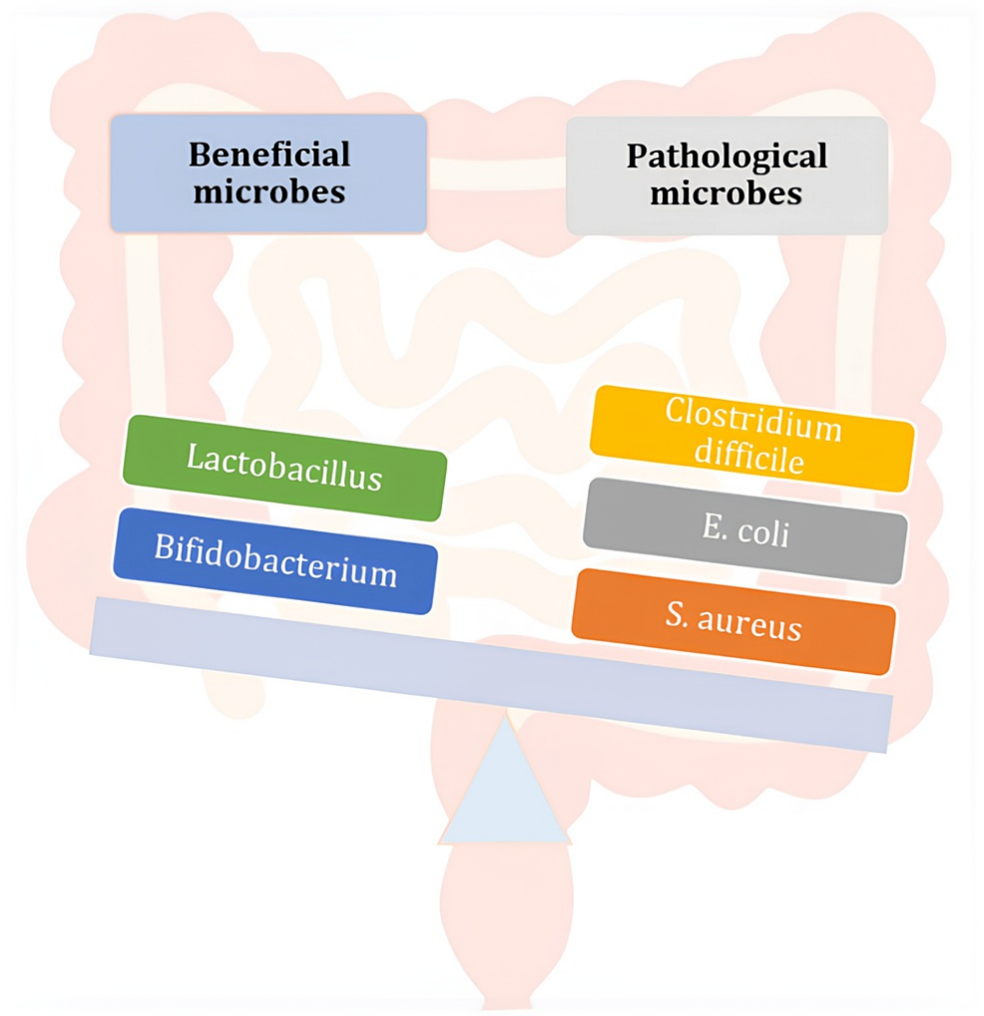
Gut dysbiosis in atopic dermatitis Image Credit: Author Pradeep Mane

The gut microbiome can impact cutaneous pathology, physiology, and immune responses by causing metastasis of gut microorganisms and their metabolites to the skin [14]. Patients with AD show microbiota abnormalities with an increased growth of Staphylococcus aureus along with a reduction in Propionibacterium, Streptococcus, and Acinetobacter. AD patients also have gut microbiota abnormalities showing a decrease in beneficial microbes like Lactobacillus and Bifidobacterium and increased Escherichia coli, S. aureus, and Clostridium difficile [11]. Figure 3 shows the gut dysbiosis in AD.

Children and infants with AD are generally treated with TCSs, antihistamines, and antibiotics. However, these medications are associated with different adverse effects, and stopping the treatment can cause the recurrence of AD symptoms. The long-term use of TCSs may trigger new onset atopic dermatitis [15]. TCSs are associated with multiple cutaneous side effects like skin atrophy, purpura, telangiectasia, hypopigmentation, acneiform eruptions, striae, and focal hypertrichosis [16]. Several studies show that probiotics supplementation may be an effective choice in reducing the incidence as well as in the treatment of atopic dermatitis [17-19]. A meta-analysis of 20 randomized controlled trials assessing probiotics alone or in combination with prebiotics in children with AD showed a significant decrease in SCORing AD (SCORAD) severity scores, suggesting a consistent pattern in alleviating AD symptoms in children without food allergies [20].

Probiotics exert beneficial effects on the host by causing an enhancement in intestinal barrier function, the suppression of pathogens, and immune modulation [11]. Probiotics enhance intestinal barrier function by promoting mucin secretion from goblet cells (MUC1, MUC2, and MUC3), limiting bacterial movement. They also increase AMP production, preventing bacterial growth, and improve tight junction stability through the upregulation of proteins like claudin-1, occludin, and zonula occludens (ZO). This, in turn, reduces the permeability of the epithelial layer to potential pathogens. Probiotics produce antimicrobial factors like AMPs, short-chain fatty acids (SCFAs), and bacteriocins to suppress or eliminate pathogens. Additionally, SCFAs such as butyrate help modulate the expression of occludin and ZO, enhancing epithelial barrier integrity. Probiotics influence the maturation of dendritic cells (DCs) by regulating their differentiation into either mature form or tolerogenic form in response to pro-inflammatory or anti-inflammatory stimuli. Probiotics balance Th1/Th2 immune response by stimulating Th1 and suppressing Th2 responses. Additionally, probiotics induce regulatory T cell (Treg) generation, fostering immune tolerance and impacting allergic diseases [11]. Table 2 shows the summary of clinical trials showing favorable outcomes with probiotics in AD.

**Table 2:** Summary of clinical trials showing favorable outcomes with probiotics in atopic dermatitis (AD) SCORAD: SCORing AD; IDQoL: Infants' Dermatitis Quality of Life; DLQ: Dermatology Life Quality; AE: adverse event

Study	Study design	Population	Probiotic strain	Duration of administration	Concomitant treatment	Efficacy results	Safety results
Pediatric AD – Lactobacillus rhamnosus studies							
Cacrucci et al. (2022) [17]	Randomized, double-blind, placebo-controlled trial	100 AD patients aged six to 36 months	L. rhamnosus GG (LGG) (1×1010 CFU)	12 weeks	Topical hydrocortisone butyrate 0.1% ointment as a rescue medication	↓SCORAD at week 12 (p<0.05), improvement in IDQoL at week 12 (p<0.05) and number of days without rescue medications were higher in the probiotic group	No AEs reported
Wu et al. (2017) [21]	Randomized, double-blind, placebo-controlled trial	Children with AD (aged four to 48 months) and SCORAD≥ 15 at enrolment.	L. rhamnosus (MP108)	eight weeks	Topical corticosteroids as needed	↓SCORAD at week eight (p<0.05) in the probiotic group	No significant difference in values of vital signs and other parameters
Schmidt et al. (2019) [22]	Randomized, double‐blind, placebo‐controlled trial,	290 infants aged eight to 14 months	A mixture of two probiotic strains (LGG and BB-12), each in a dose of 109 CFU	six months	Did not mention	Lower incidences of eczema (p=0.036) were observed in the probiotic group as compared to the placebo group	Did not mention
Viljanen et al. (2005) [23]	Randomized, double-blind, placebo-controlled trial	230 children aged 1.4 to 11.9 months with AD	LGG (ATCC 53103) or a mixture of four probiotics or placebo	four weeks	Did not mention	↓SCORAD in IgE-sensitized infants at week four (p=0.036) in the probiotic group	Did not mention
Kalliomaki et al. (2001) [18]	Randomized, double-blind, placebo-controlled trial	Prenatally to mothers who had a family history of atopic disease and postnatally to either infants or breastfeeding mothers	LGG ATCC 53103 (2×1010)	Prenatally - two to four weeks before expected delivery, postnatally - six months	Did not mention	At two years, the frequency of atopic eczema in the LGG group (15/64 (23%)) was half that of the placebo group (31/68 (46%)) (p=0.008)	Did not mention
Kalliomaki et al. (2007) [24]	Randomized, double-blind, placebo-controlled trial	Prenatally to mothers who had a family history of atopic disease and postnatally to either infants or breastfeeding mothers	LGG ATCC 53103 (2×1010)	Prenatally - two to four weeks before expected delivery, postnatally - six months	Did not mention	At seven years, the overall risk for developing atopic eczema during the first seven years of life in children was significantly decreased in the LGG group than in the placebo group (42.6% vs. 66.1%)	Did not mention
Rautava et al. (2002) [19]	Double-blind, placebo-controlled trial	62 mothers with a family history of atopic diseases	LGG (ATCC 53103); 2 × 1010 CFU/d	Four weeks prenatal, three months postnatal (mothers only)	Did not mention	Infants whose mothers received probiotics had significantly reduced risk of developing AD during the first two years of life in comparison with infants whose mothers received placebo (15% and 47%, respectively (p=.0098)	No AEs
Pediatric AD - other probiotics							
Navarro-López et al. (2018) [25]	Randomized, double-blind, placebo-controlled intervention trial	50 children aged four to 17 years with moderate AD	Mixture of Bifidobacterium lactis CECT 8145, Bifidobacterium longum CECT 7347, and Lactobacillus casei CECT 9104	12 weeks	Topical methylprednisolone aceponate, moisturizer, and oral antihistamine	↓SCORAD at week 12 in the probiotic group (p<0.001), Use of topical corticosteroids to treat flares decreased significantly in the probiotic group as compared to the placebo group (p<0.003)	No relevant AEs
de Andrade et al. (2022) [26]	Randomized, double-blind placebo-controlled trial	60 patients aged between six months and 19 years with mild, moderate, or severe AD	Mixture of L. rhamnosus HN001; Lactobacillus acidophilus; Lactobacillus paracasei Lcp-37; and B. lactis HN019	Six months	Montelukast, topical immunosuppressants, oral corticosteroids, topical corticosteroids, anti-histamines, and moisturizers	↓SCORAD at six months in the probiotic group (p=0.03). Probiotics group required topical immunosuppressant less frequently at six and nine months (p<0.05)	Nausea, abdominal pain, and worsened pruritic episodes (no severe AEs)
Adult AD - other probiotics							
Iemoli et al. (2012) [27]	Randomized, double-blind, placebo-controlled trial	48 adult patients with AD (randomization ratio 2:1)	Combination of (Lactobacillus salivarius LS01 and Bifidobacterium breve BR03), each with a dose of 1 × 109 CFU/g in maltodextrin, twice daily	12 weeks	Oral antihistamines or emollient cream	↓SCORAD (p<0.0001)and improvement in DLQ index (p=0.021) in the probiotic group, Probiotics reduced microbial translocation (p=0.050), immune activation (p<0.001), and improved Th17/Treg (p=0.029) and Th1/Th2 (p=0.028) ratios.	No significant AEs
Drago et al. (2011) [28]	Randomized, double-blind, placebo-controlled trial	38 patients with moderate/severe AD aged 18 to 46 years	L. salivarius LS01 at a dose of 1 x 109 CFU/g, twice daily	16 weeks	Oral antihistamines or emollient cream	↓SCORAD (p<0.0001) and improvement in DLQ index (p=0.021) at week 16 in the probiotic group	No significant AEs
Prakoeswa et al. (2022) [29]	Randomized, double-blind, placebo-controlled trial	30 adults with mild and moderate AD	Probiotic microencapsulation of Lactobacillus plantarum (LP) IS-10506 (2 × 1010 CFU/day)	Eight weeks	Did not mention	Significant decrease in the SCORAD index in the probiotic group as compared to the placebo group	No significant side effects

Dose of the probiotic (LGG)

The usual dose of LGGis 5-10 × 109 CFU/d for children and 10-20 × 109 CFU/d for adults [30,31].Clinical studies on AD using LGG in a dose of 1×1010 CFU/day showed significant efficacy outcomes in pediatric populations [17,18,24].

Table 3 lists the recommendations for the use of oral probiotics in the treatment of AD.

**Table 3:** Recommendations on the use of oral probiotics in the treatment of atopic dermatitis (AD) LoE: level of evidence; LoA: level of agreement

Sr. No.	Recommendations
1.	Improvement in nutritional status, immunomodulatory properties, and beneficial effects on the gastrointestinal tract and skin, support the use of probiotics in patients with AD (LoE=2, LoA=88.88%).
2.	Probiotics can be used in the prevention as well as the treatment of AD (LoE=2, LoA=88.88%).
3.	Oral probiotics in the right concentration as an adjuvant treatment are effective in the management of AD and can be considered as steroid sparing strategy or maintenance therapy in high-risk cases with flares (LoE=2, LoA=77.77%).
4.	Probiotics can be prescribed to AD patients for the duration of at least eight to 12 weeks (LoE=2, LoA=77.77%).
5.	Usual probiotic dose in pediatric populations is 5-10 × 109 CFU/day (LoE=2, LoA=88.88%).
6.	Usual probiotic dose in adult populations is 10-20 × 109 CFU/day (LoA=77.77%).

However, this study has several limitations. The sample size of 175 dermatologists may not fully represent all regions of India. The study lacked direct patient outcomes and longitudinal data, limiting conclusions about long-term efficacy and safety. Additionally, relying on the published literature means unpublished data can alter current recommendations.

## Conclusions

The prevalence of AD is rising in the Indian population, presenting significant challenges in its management due to the need for prolonged treatment. This KAP survey offers valuable insights into the real-life application of probiotics in managing AD. The survey findings indicate that probiotics, particularly LGG, can be beneficial as an adjuvant therapy, helping to manage AD and associated flare-ups when used alongside traditional treatments.

Probiotics demonstrate potential in improving nutritional status, enhancing immune responses, and benefitting gastrointestinal and skin health, which supports their use in AD management. While further research is needed to confirm these benefits and optimize treatment protocols, this survey underscores the promising role of probiotics as part of a comprehensive treatment strategy for AD.
